# Minimum Effective Dose of Cattle and Sheep BSE for Oral Sheep Infection

**DOI:** 10.1371/journal.pone.0151440

**Published:** 2016-03-11

**Authors:** Gillian McGovern, Stuart Martin, Martin Jeffrey, Glenda Dexter, Steve A. C. Hawkins, Sue J. Bellworthy, Lisa Thurston, Lynne Algar, Lorenzo González

**Affiliations:** 1 Animal and Plant Health Agency (APHA-Lasswade), Pentlands Science Park, Penicuik, Midlothian, EH26 0PZ, United Kingdom; 2 APHA-Weybridge, Addlestone, Surrey, KT15 3NB, United Kingdom; Creighton University, UNITED STATES

## Abstract

The minimum dose required to cause infection of Romney and Suffolk sheep of the ARQ/ARQ or ARQ/ARR prion protein gene genotypes following oral inoculation with Romney or Suffolk a sheep Bovine spongiform encephalopathy (BSE)-derived or cattle BSE-derived agent was investigated using doses ranging from 0.0005g to 5g. ARQ/ARQ sheep which were methionine (M) / threonine (T) heterozygous or T/T homozygous at codon 112 of the *Prnp* gene, dosed ARQ/ARR sheep and undosed controls did not show any evidence of infection. Within groups of susceptible sheep, the minimum effective oral dose of BSE was found to be 0.05g, with higher attack rates following inoculation with the 5g dose. Surprisingly, this study found no effect of dose on survival time suggesting a possible lack of homogeneity within the inoculum. All clinical BSE cases showed PrP^d^ accumulation in brain; however, following cattle BSE inoculation, LRS involvement within Romney recipients was found to be significantly lower than within the Suffolk sheep inoculated group which is in agreement with previous reports.

## Introduction

Transmissible spongiform encephalopathies (TSEs) or prion diseases are chronic, fatal, neurodegenerative diseases affecting humans and animals that may be acquired following oral exposure to the infectious agent. Thus, bovine spongiform encephalopathy (BSE) and variant Creutzfeldt-Jakob disease (vCJD) occurred as a result of the consumption of prion contaminated meat and bone meal [1] and BSE infected meat [2, 3], respectively. Similarly, kuru is associated with cannibalistic rituals [4] and chronic wasting disease and scrapie are also thought to be acquired orally [5, 6].

Sheep orally infected with cattle BSE show some features which are in common with naturally occurring cattle BSE, such as the accumulation of disease associated prion protein (PrP^d^) in the brain [7] and the biochemical properties of its protease-resistant core [8]. However, BSE-infected sheep show a much wider distribution of PrP^d^ and infectivity [9, 10] than cattle BSE [11–13], particularly within lymphoreticular system (LRS) tissues, and in this respect ovine BSE resembles natural scrapie.

Exposure of sheep to BSE-contaminated feed, together with the wide organ distribution of infectivity in BSE-infected sheep, raised concerns that BSE could be contagious and, like scrapie, have been maintained in the UK sheep flock while being masked by endemic scrapie. Certainly, a recent report on a large, long-standing experiment has shown that ovine BSE is contagious amongst sheep, albeit with a low degree of transmissibility [14]. One caveat of that experiment was that the oral dose used to generate the parental flock was relatively high (5g of cattle BSE); this gave rise to considerations about lower doses being still infectious while resulting in longer survival times that could have allowed a more efficient natural transmission of BSE.

Therefore, determination of the minimum effective oral dose and the effect of dose on attack rate (AR) and survival time (ST) are essential for assessing the potential risk of BSE occurring in the UK sheep flock. In the studies reported here, we aimed to identify differential features between ovine BSE resulting from oral infection with either cattle- or sheep-derived BSE in sheep of two different breeds but the same susceptible prion protein gene (*PRNP*) genotype, and to assess the effect of dose on such features. Partial and preliminary results from these experiments, involving a limited number of sheep and dealing with the differential immunohistochemical (IHC) and biochemical features of the disease in comparison with natural scrapie, have been reported previously [15]. The present report provides the final overall outcome of the experiments and concentrates on clinical parameters, AR and ST, and on tissue distribution of PrP^d^.

## Materials and Methods

All animal experiments were approved by the local ethics committee of the Animal and Plant Health Agency (formerly Veterinary Laboratories Agency) and carried out in accordance with the Animals (Scientific Procedures) Act 1986 under Home Office license numbers 70/5780 and 70/5781. Sheep were sourced from a New-Zealand-derived, classical TSE-free flock (ARSU flock, APHA/Defra, UK); all male sheep were castrated.

In the first experiment, 50 Romney sheep (G1; 19 male and 31 female) and 50 Suffolk sheep (G2; 37 male and 13 female), (G2) of the ARQ/ARQ *PRNP* genotype (A, alanine, R, arginine and Q, glutamine at codons 136, 154 and 171 of PrP, respectively) were orally dosed at three and six months of age, respectively, with the same cattle-derived BSE inoculum (brain pool from clinically affected, BSE-confirmed cattle), as used in a previously described experiment [10]. Each group was divided into five subgroups of 10 sheep each, which received 10-fold dilutions (5g to 0.0005g) of such inoculum and another 10 sheep of each breed (8 male and 2 female Suffolk sheep and 4 male and 6 female Romney sheep) were kept as age-matched, undosed controls (Table 1).

**Table 1:** Experimental design.

	G1	G2	G3	G4	G5	G6	G7	G8	
Dosing	C to R	C to S	R^1^ to R	R^1^ to S	R^1^ to R^QR^	S^1^ to R	S^1^ to S	R^2^ to R	Total
5g	10	10 (3^#^)	5	5 (1^#^)	5	5	5	5	**50 (4**^#^**)**
0.5g	10 (1*)	10 (3^#^)	5	5 (1^#^)	5 (1*)	5	5 (1^#^)	5	50 (2*,5^#^**)**
0.05g	10	10 (2^#^)	5	5 (1^#^)	5 (1*)	5	5 (3^#^)	5 (1*)	50 (2*,6^#^**)**
0.005g	10	10 (2^#^)	5	5 (1^#^)	5 (1*)	5	5 (2^#^)	5 (1*)	50 (2*,5^#^**)**
0.0005g	10 (1*)	10 (1*,1^#^)	5	5 (1^#^)	5	5	5 (2^#^)	5	50 (2*,4^#^**)**
Undosed	10	10 (3^#^)	0	5 (2^#^)	5	5	5 (1^#^)	5	**45 (6**^#^**)**
**Total**	60 (2*)	60 (1*,14^#^**)**	**25**	**30 (7**^#^**)**	30 (3*)	**30**	**30 (9**^#^**)**	30 (2*)	295 (8*,30^#^**)**

G1 to G8, experimental groups as defined in the text. C, cattle BSE-derived inoculum; R^1^, first passage Romney sheep BSE-derived inoculum; S^1^, first passage Suffolk sheep BSE-derived inoculum; R^2^, second passage Romney sheep BSE-derived inoculum. R, Romney sheep recipients; S, Suffolk sheep recipients; R^QR^, ARQ/ARR Romney sheep recipients (all others are ARQ/ARQ).

*, intercurrent deaths;

^#^, M_112_T or T_112_T Suffolk sheep.

In the second experiment, three sheep BSE-derived inocula were used to orally challenge groups of 25 six month-old sheep of the same two breeds; each group was divided into subgroups of five sheep each which received one of the five different doses (5g to 0.0005g). The experimental groups were: 1) ARQ/ARQ Romney (G3; 25 female), ARQ/ARQ Suffolk (G4; 13 male and 12 female) and ARQ/ARR Romney sheep (G5; 25 male), which received a first passage Romney sheep BSE-derived inoculum (brain pool from clinically affected sheep of the experiment reported by McGovern *et al*. [10]), 2) ARQ/ARQ Romney (G6; 10 male and 15 female), and ARQ/ARQ Suffolk sheep (G7; 14 male and 11 female), which were dosed with a first passage Suffolk sheep BSE-derived inoculum (from the same experiment as indicated above) and 3) a further group of ARQ/ARQ Romney sheep (G8; 12 male and 13 female), which received a second passage Romney sheep BSE-derived inoculum (brain pool from clinically affected sheep of G3 above). Ten ARQ/ARQ Romney sheep (4 male and 6 female), and Suffolk sheep (8 male and 2 female) and 5 ARQ/ARR male Romney sheep remained as age-matched, undosed controls for the different experimental groups (Table 1). All inocula were prepared in 10 ml of phosphate saline buffer and administered by syringe in the back of the throat to ensure full swallowing.

All experimental groups were kept separate throughout the experiments in purpose built accommodation and husbandry and handling practices were set up to minimize chances of cross-contamination between groups. Bedding consisted of wood shavings which were replenished / removed as necessary to ensure dry bedding was maintained and the sheep were offered forage *ad libitum* supplemented with an appropriate quantity of concentrate. Sheep were vaccinated with heptavac P and treated regularly with doramectin, in addition to quarterly foot trim and bathing, and twice yearly shearing. Sheep were subject to clinical monitoring including standard daily observations, monthly bodyweight measurement and condition scoring, and monthly scratch response testing, and were euthanized using sodium barbiturate when they reached a clinical end point with signs characteristic of a TSE, as previously reported [16] or at the termination of the experiments. Some sheep died spontaneously (n = 2) or were euthanized (n = 6) as a result of intercurrent disease (Table 1) and were excluded from the study either because they died earlier than the average survival time of their corresponding subgroup or, in the case of subgroups with zero attack rate, because they survived for less than 1,500 days after dosing.

A retrospective, full *PRNP* open reading frame genotyping was conducted at the end of the studies and some Suffolk sheep (Table 1) were found to be MT or TT at codon 112 of *PRNP* (M, methionine and T threonine); they were not considered in the calculations of ARs and STs in view of the reported resistance of such sheep to oral BSE [10, 17].

At post-mortem, samples of brain, enteric nervous system (ENS; jejunum and ileum), cranial mesenteric ganglion (CMG) and selected LRS tissues (retropharyngeal, prescapular, distal jejunal and prefemoral lymph nodes, palatine tonsil, spleen and jejunal and ileal Peyer's patches) were collected and placed into 10% formaldehyde for IHC examinations. Following paraffin wax embedding, 4μm tissue sections were cut and immunolabelled using monoclonal PrP antibody R145 (APHA, Weybridge, UK) by procedures described elsewhere [18].

## Results

Once sheep dying from intercurrent disease are excluded as explained above (n = 8, with survival times of 591±224 days post-inoculation (dpi; average±standard deviation), evidence of BSE–both clinical disease and IHC detection of PrP^d^–was completely absent from the following sheep and experimental groups: 1) the 22 dosed ARQ/ARR sheep from G5 culled at 2475±413 dpi, 2) the 24 M_112_T or T_112_T dosed Suffolk sheep from G2, G4 and G7 culled at 2463±287 dpi, 3) the 45 undosed controls culled at 2654±118 days of age and 4) 78 ARQ/ARQ sheep from any groups dosed with 0.005g or 0.0005g and culled at 2458±235 dpi.

Therefore, the analysis of differences in AR is circumscribed to 118 sheep of the 295 of the whole experimental set up, that is, those dosed with 5g, 0.5g or 0.05g, which were not excluded because of intercurrent disease or *PRNP* genotype issues. Statistical analyses of differences in AR between the different groups were done by Fisher's exact test. When comparing sheep inoculated with the different inocula and considering the three doses together, no differences were observed between cattle- (21/51, 41.2%) and the three different sheep-derived BSE inocula (Romney 1^st^ passage 9/27, 33.3%; Romney 2^nd^ passage 5/14, 35.7%; Suffolk 1^st^ passage 16/26, 61.6%); the only comparison that yielded nearly significant differences was between sheep dosed with Suffolk and Romney 1^st^ passage inocula (P = 0.056). Equally, no differences between inocula were observed when considering the three doses separately (Table 2). No differences in AR were found between the two recipient breeds, with Romney and Suffolk sheep showing similar overall figures (30/73, 41.1% and 21/45, 46.7%, respectively); this absence of difference was maintained when the analyses were split by dose (Table 2).

**Table 2:** Attack rates of experimental oral BSE according to inoculum source and dose and recipient host.

			Dose		
Group	Description	5g	0.5g	0.05g	Total
G1	C to R	8/10 (80.0)	3/9 (33.3)	1/10 (10.0)	12/29 (41.4)
G2	C to S	6/7 (85.7)	1/7 (14.3)	2/8 (25.0)	9/22 (40.9)
G3	R^1^ to R	2/5 (40.0)	2/5 (40.0)	0/5 (0.0)	4/15 (26.7)
G4	R^1^ to S	4/4 (100)	1/4 (25.0)	0/4 (0.0)	5/12 (41.7)
G6	S^1^ to R	4/5 (80.0)	4/5 (80.0)	1/5 (20.0)	9/15 (60.0)
G7	S^1^ to S	5/5 (100.0)	1/4 (25.0)	1/2 (50.0)	7/11 (63.6)
G8	R^2^ to R	4/5 (80.0)	1/5 (20.0)	0/4 (0.0)	5/14 (35.7)
All groups	Total	33/41 (80.5) A	13/39 (33.3) B***	5/38 (13.2) B***	51/118 (43.2)
G1+2	Cattle inoculum	14/17 (82.4) A	4/16 (25.0) B**	3/18 (16.7) B***	21/51 (41.2)
G3+4	Romney inoculum 1st	6/9 (66.7) A	3/9 (33.3) AB	0/9 (0.0) B**	9/27 (33.3)
G8	Romney inoculum 2nd	4/5 (80.0) A	1/5 (20.0) AB	0/4 (0.0) B*	5/14 (35.7)
G6+7	Suffolk inoculum 1st	9/10 (90.0) A	5/9 (55.6) AB	2/7 (28.6) B*	16/26 (61.6)
G1+3+6+8	Romney recipients	18/25 (72.0) A	10/24 (41.7) B*	2/24 (8.3) B***	30/73 (41.1)
G2+4+7	Suffolk recipients	15/16 (93.8) A	3/15 (20.0) B***	3/14 (21.4) B***	21/45 (46.7)

G1 to G8, experimental groups as defined in the text. C, cattle BSE-derived inoculum; R^1^, first passage Romney sheep BSE-derived inoculum; S^1^, first passage Suffolk sheep BSE-derived inoculum; R^2^, second passage Romney sheep BSE-derived inoculum. R, Romney sheep recipients; S, Suffolk sheep recipients. Results expressed as BSE positive/challenged (%); note that this table does not include the intercurrent deaths, the ARQ/ARR Romney or the M_112_T or T_112_T Suffolk sheep recipients, the animals dosed with 0.005g or 0.0005g of inoculum, or the undosed controls. Statistical analyses of differences between doses are given for the total of sheep and for the different inocula and sheep recipients and are indicated by capital letters: values with no letter in common (reading by rows) are significantly different in the Fisher's exact test with

*, P<0.05

**, P<0.01 or

***, P<0.001.

Infectious titers of the four different inocula were calculated using the Reed-Muench formula [19]. These, expressed as 1x (oral) ID_50_/g, were estimated as 1x10^-0.14^ for the cattle inoculum, 10^−0.20^ for the Romney sheep-derived inoculum (identical for first and second passage) and 10^0.46^ for the Suffolk sheep-derived inoculum.

ARs were clearly influenced by the dose of inoculum administered, so that the 5g dose produced higher attack rates than the 0.5g and, particularly, the 0.05g doses. This was true when the comparison was made for all 118 sheep (5g, 33/41 [80.5%]; 0.5g, 13/39 [33.3%]; 0.05g, 5/38 [13.2%]; P<0.0001 in the two comparisons), when the analysis was carried out for the two recipient breeds (combined inocula) and within sheep dosed with cattle inoculum (Table 2). In the case of sheep dosed with the three different ovine BSE sources, those dosed with 0.5g showed non-significantly lower attack rates than those dosed with 5g. Differences were more subtle when comparing the 0.5g and the 0.05g, insofar as they were not significant in any of the analyses performed (Table 2). If in view of the absence of any difference, the 0.5g and 0.05g groups are merged together, then the differences between the 5g group and the merged group become significant in all analyses (results not shown but can be inferred from data presented in Table 2).

**Fig 1:**
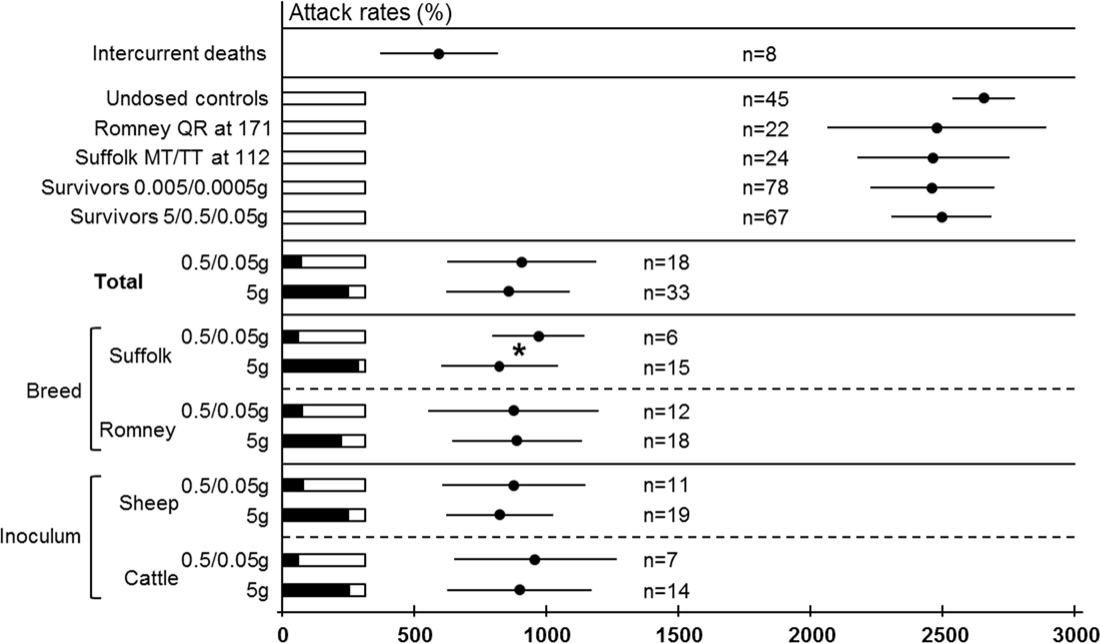
Graphical and schematic representation of the outcome of the experiments in terms of attack rates and survival times. Attack rates (%), black bars inside white boxes, with actual values next to them. Survival times are indicated as average±SD dpi (age in case of undosed controls). Note that despite the marked differences in AR between the 5g and the 0.5/0.05g doses, STs are very similar with the only significant difference marked as * (for details refer to text).

**Fig 2:**
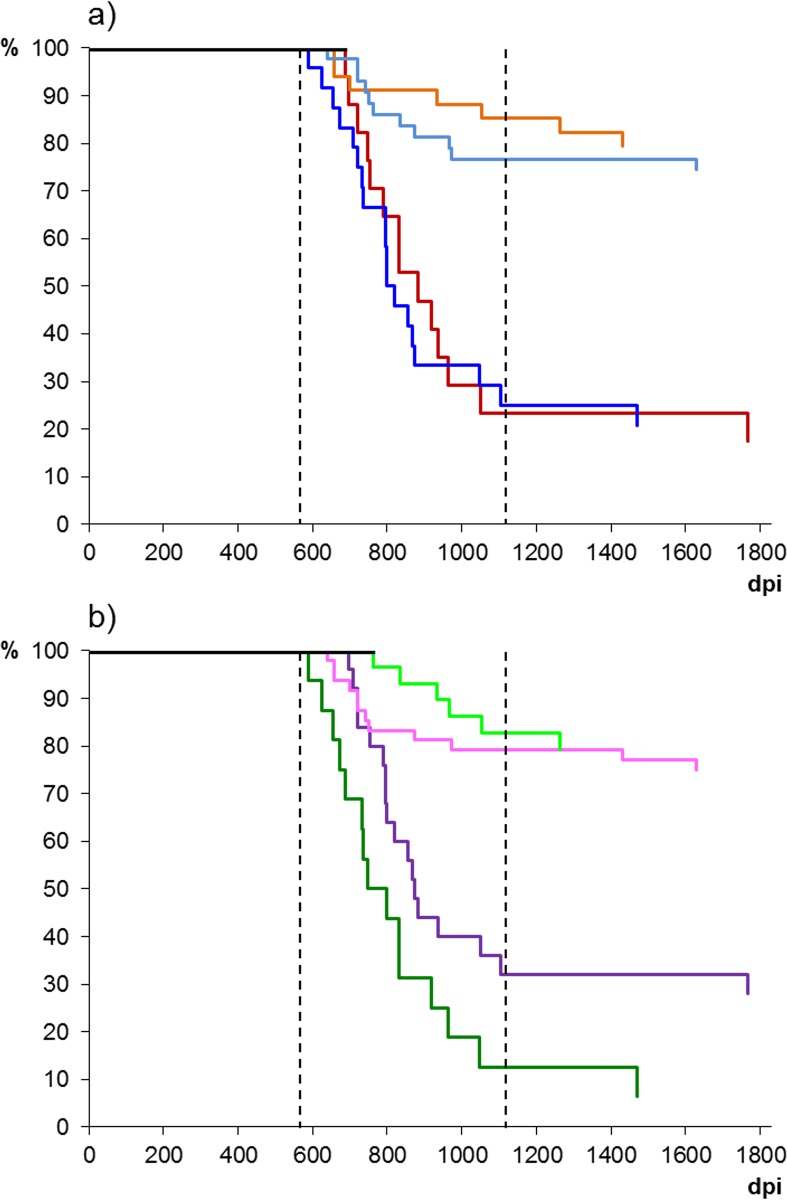
Survival curves of sheep that developed IHC-confirmed BSE according to: a) source and dose of inoculum: dark blue, sheep inocula at 5g dose; light blue, sheep inocula at 0.5/0.05g dose; red, cattle inoculum at 5g dose; orange, sheep inocula at 0.5/0.05g dose. b) recipient breed and dose of inoculum: dark green, Suffolk sheep dosed with 5g; light green, Suffolk sheep dosed with 0.5/0.05g; purple, Romney sheep dosed with 5g; pink, Romney sheep dosed with 0.5/0.05g. Note that in both graphs, most sheep succumbed between ~600 and 1,150 days regardless of dose received and that the few sheep that survived for longer than ~1,150 days did so also regardless of the dose they received.

Comparative analyses of STs were restricted to the 51 sheep developing clinical BSE confirmed by IHC examination at post-mortem; the other 67 sheep considered in the AR were culled in the absence of clinical disease and of IHC evidence of infection at 2495±189 dpi. Since the differences in ARs were minimal and the number of affected sheep small, the data from the 0.5g and 0.05g doses were pooled together; for the same reasons the three sheep-derived BSE inocula were merged into one. Because the data were in most cases not normally distributed, the analyses were done by the non-parametric Mann-Whitney test. No differences in ST were observed between sheep given cattle- or sheep-derived BSE, either in the overall analysis (918±279 dpi v’s 842±227 dpi, respectively) or when the data were split by the two dose groups (statistical analyses not shown but can be inferred from data presented in Figs 1 and 2). Survival times were also similar for the two recipient breeds regardless of whether the analysis was done for all doses combined (881±274 dpi for Romney vs 863±216 for Suffolk sheep), or separately within the 5g and 0.5/0.05g doses, with the exception indicated below (statistical analyses not shown but can be inferred from data presented in Figs 1 and 2).

The dose of inoculum did not have a relevant impact on the STs of the affected sheep. Thus, in the overall analysis, sheep dosed with 5g had a marginally shorter survival time than those given 0.5g/0.05g (855±234 dpi and 906±281 dpi, respectively; P = 0.7). This lack of any difference was also patent when performing the analyses separately within the cattle and sheep derived inocula or within the Romney recipients (statistical analyses not shown but can be inferred from data presented in Figs 1 and 2). The only significant difference was found when analyzing the group of Suffolk sheep recipients in which sheep dosed with 0.5g/0.05g (n = 6) showed longer survival times (969±176 dpi) than those dosed with 5g (n = 15; 820±221 dpi; P = 0.04).

All 51 clinical BSE cases were positive by IHC in brain and, with two exceptions, showed widespread PrP^d^ accumulation in the ENS and CMG. The exceptions were two Suffolk sheep, one dosed with Suffolk-derived inoculum, which was negative in the jejunal ENS and in the CMG, and another dosed with cattle BSE, which was negative in the jejunal ENS. All these 51 sheep were also positive in variable proportions of the LRS tissues examined. Thus, sheep dosed with 5g of the different sheep-derived BSE inocula were positive in ~79% (G7), ~83% (G8), ~92% (G6), ~93% (G3) or 100% (G4), while all sheep dosed with 0.5g or 0.05 were positive in all LRS tissues examined (Table 3). Following cattle BSE inoculation, LRS involvement within the Romney recipients (G1; ~76% for the three doses combined) was found to be significantly lower (P<0.0001) than within the Suffolk sheep (G2; ~98% for the three doses combined) inoculated group (Table 3). The IHC features of PrP^d^ accumulation in the different tissues and systems of some of the clinically affected sheep of these experiments have been reported previously [15]; those that were not included in that report showed the same, BSE-characteristic features.

**Table 3:** Frequency of accumulation of PrP^d^ in the LRS tissues of BSE positive sheep.

			Dose		
Group	Description	5g	0.5g	0.05g	Total
G1	C to R	50/71 (70.4)	23/27 (85.2)	5/8 (62.5)	78/106 (75.6)
G2	C to S	95/95 (100.0)	9/9 (100.0)	16/18 (88.9)	120/122 (98.4)
G3	R^1^ to R	13/14 (92.9)	14/14 (100.0)	n/a	27/28 (96.4)
G4	R^1^ to S	24/24 (100.0)	7/7 (100.0)	n/a	31/31 (100.0)
G6	S^1^ to R	11/12 (91.7)	12/12 (100.0)	3/3 (100.0)	26/27 (96.3)
G7	S^1^ to S	15/19 (78.9)	3/3 (100.0)	3/3 (100.0)	21/25 (84.0)
G8	R^2^ to R	10/12 (83.3)	3/3 (100.0)	n/a	13/15 (86.7)

G1 to G8, experimental groups as defined in the text. C, cattle BSE-derived inoculum; R^1^, first passage Romney sheep BSE-derived inoculum; S^1^, first passage Suffolk sheep BSE-derived inoculum; R^2^, second passage Romney sheep BSE-derived inoculum. Results expressed as positive LRS tissues/total examined (%) for each dose group and for the total of all doses combined. The only significant differences in the Fisher's exact test (P<0.001) are between Romney and Suffolk sheep challenged with 5g dose and for all doses combined.

## Discussion

In terms of influence of the sheep host *PRNP* genotype on the susceptibility to TSEs, these experiments have confirmed previous reports and have brought up some new insights. On one hand the resistance of codon 112 threonine homo- and hetero-zygotes to oral BSE, confirms the findings of previous studies with respect to cattle-derived inocula [10, 17] and show that such resistance is maintained for sheep-derived inocula. On the other hand, allowing for the relatively small number of sheep examined, the resistance of ARQ/ARR sheep to sheep-derived BSE contrasts with the reported sensitivity of sheep of this genotype to oral dosing with sheep scrapie with a same 5g dose [20]. It is unlikely that this discrepancy is due to scrapie being more infectious for sheep than BSE, since other experiments have shown that, for ARR/ARR sheep intracerebrally challenged, the BSE agent is more infectious [21] than the scrapie agent [22]. A more plausible explanation is the difference in age at challenge between this experiment (6 months) and the one reported by González *et al*. [20], in which pre-weaned lambs (10–15 days old) were inoculated; this age-related susceptibility of sheep to BSE would be in agreement with previously reported experimental evidence [23]. Also in agreement with previous studies [10, 23, 24] would be the lack of age-related susceptibility for sheep orally dosed at three or six months of age.

Romney sheep dosed with cattle BSE showed a lower involvement of LRS tissues at clinical end point compared to Suffolk sheep dosed with the same BSE source, which is in agreement with a previous study in which sheep of the same two breeds were killed sequentially at different time points after oral inoculation [10]. However, no differences in LRS involvement between the two breeds were noted when the animals were challenged with sheep-derived BSE, suggesting some sort of adaptation of the BSE agent to replicate and accumulate in the LRS tissues of Romney sheep after sheep passage. This was actually the only effect observed that could be attributable to the source of the inoculum since in terms of minimum effective dose, ARs and STs cattle-derived BSE and the three sheep-derived BSE inocula behave similarly. Therefore, the alleged increased pathogenicity of sheep BSE compared to cattle BSE for transgenic mice expressing bovine [25], porcine [26] or human [27, 28] PrP has not been corroborated in the present study, as far as sheep recipients is concerned.

The studies presented in this report indicate that the minimum effective oral dose of BSE for ARQ/ARQ sheep is 0.05g (50mg), with an overall attack rate of ~13% (~17% for cattle- and 10% for sheep-derived inoculum). This figure contrasts with the minimum effective dose of ovine BSE for sheep of the same genotype on intracerebral inoculation, which was worked out as 0.00005g (0.05mg) tissue equivalent, with a 60% attack rate [29]. A comparison between the two figures would suggest that the efficiency of the intracerebral route is around 10^3^ times higher than that of oral dosing. In terms of ID_50_, the results from the experiments of this report provide an oral dose figure of BSE for sheep of 10^−0.05^, with variations between 10^-.020^ for Romney sheep inoculum and 10^0.46^ for Suffolk sheep inoculum. To make these figures more comparable with those obtained in the intracerebral experiment, where Romney sheep were challenged with sheep-derived BSE [29], the ID^50^ was calculated for Romney recipients orally dosed with sheep-derived inoculum, that is, G3, 6 and 8 of the present report. This calculation provided a figure of 10^0.29^, approximately 10^5^ times lower than the 10^5.4^ obtained in the intracerebral experiment. Whatever the calculation used, and allowing for possible differences in infectious titres between the inocula used in these experiments, the markedly different efficiencies between the oral and intracerebral routes 1) could explain why a proportion of ARR/ARR and ARQ/ARR sheep succumb to intracerebral BSE challenge [21, 29] while they are resistant to orally-administered BSE [10 and G5 in this experiment] and 2) highlight the need of a cautious interpretation of data obtained from intracerebral experiments when performing risk analyses and assessments.

Perhaps slightly surprising is the lack of effect of dose on survival time, which nevertheless has been documented in other studies [23]. One possible explanation could be that the infectious agent is not homogeneously distributed in the inoculum. Thus, highly concentrated doses, such as 5g, would produce high numbers of infectious aliquots (high attack rate) while more diluted doses, such as 0.5g or 0.05g, would produce lower number of infectious aliquots (lower attack rate) but of a similar infectiousness giving rise to similar survival times. Conversely, if the infectious agent was homogeneously distributed in the inoculum, it would be anticipated that highly concentrated inocula would contain high numbers of highly infectious aliquots resulting in high attack rates and short incubation periods, while more diluted inocula would produce high numbers of less infectious aliquots resulting in similar attack rates with longer incubation periods, which was not the case in this study. With a similar rationale, an alternative explanation could perhaps arise from the mechanism of uptake of infectious agent from the digestive tract. In this case, it could be argued that highly concentrated inocula could result in the absorption of infectious units by most exposed animals (high attack rate), while diluted inocula would lead to uptake of infectious units by fewer animals (lower attack rate); in both cases, the absorbed infectious units could amplify with similar efficiency leading to clinical disease with similar incubation periods. Whichever the case, heterogeneous distribution of the infectious agent in BSE contaminated foodstuffs or mechanism of absorption in the digestive tract, these could explain why, during the BSE epidemic in the UK, the intra-herd incidence was usually low or very low despite generalized consumption of such feeds. Similarly, it could also explain why, despite what it is assumed to be a high or generalized exposure of people with susceptible *PRNP* genotype to BSE contaminated meat, the actual number of vCJD cases has been lower than most predictions.

Finally, the results of these experiments provide reassurance on the outcome of the sheep BSE natural transmission study previously reported [14]. The low efficiency of maternal transmission observed in that experiment was interpreted, at least in part, as due to the fact that incubation periods of the orally dosed dams were relatively short, which meant that in most cases infected ewes only lambed once. The experiments reported here suggest that the incubation periods would have been similarly short if the ewes had been infected with lower doses than the 5g used and that, consequently, the efficiency of natural BSE transmission from dam to offspring would be similarly low regardless of the infectious dose to which the ewes are exposed. Therefore, the present results support the notion that if UK sheep were exposed to BSE-contaminated feedstuffs (as is generally accepted) and natural transmission occurred to offspring of infected dams (as previously demonstrated), it is most likely that BSE infection would not have been maintained within the National flock and would have by now disappeared.
